# Clinical Use of Improved Diagnostic Testing for Detection of Prion Disease

**DOI:** 10.3390/v13050789

**Published:** 2021-04-28

**Authors:** Mark P. Figgie, Brian S. Appleby

**Affiliations:** 1 Department of Neurology, University Hospitals Cleveland Medical Center, Case Western Reserve University, Cleveland, OH 44106, USA; mark.figgie2@uhhospitals.org; 2 National Prion Disease Pathology Surveillance Center, Case Western Reserve University, Cleveland, OH 44106, USA

**Keywords:** prion disease, Creutzfeldt–Jakob disease (CJD), diagnostic testing, atypical prion disease, chronic wasting disease, RT-QuIC, alpha-synuclein, tau, 14-3-3

## Abstract

Prion diseases are difficult to recognize as many symptoms are shared among other neurologic pathologies and the full spectra of symptoms usually do not appear until late in the disease course. Additionally, many commonly used laboratory markers are non-specific to prion disease. The recent introduction of second-generation real time quaking induced conversion (RT-QuIC) has revolutionized pre-mortem diagnosis of prion disease due to its extremely high sensitivity and specificity. However, RT-QuIC does not provide prognostic data and has decreased diagnostic accuracy in some rarer, atypical prion diseases. The objective of this review is to provide an overview of the current clinical utility of fluid-based biomarkers, neurodiagnostic testing, and brain imaging in the diagnosis of prion disease and to suggest guidelines for their clinical use, with a focus on rarer prion diseases with atypical features. Recent advancements in laboratory-based testing and imaging criteria have shown improved diagnostic accuracy and prognostic potential in prion disease, but because these diagnostic tests are not sensitive in some prion disease subtypes and diagnostic test sensitivities are unknown in the event that CWD transmits to humans, it is important to continue investigations into the clinical utility of various testing modalities.

## 1. Introduction

Prion diseases are a group of invariably fatal, rapidly progressive neurodegenerative diseases caused by the aggregation of misfolded disease associated prion protein (PrP^Sc^). As a group, prion diseases share the same pathological mechanism, but have various clinical distinctions that can make them difficult to recognize and diagnose. In this review article, we will discuss the clinical utility of currently available diagnostic testing with a focus on recently developed tests with high specificity for prion disease as well as diagnosing atypical prion diseases. We will also summarize recent investigations into the diagnostic and prognostic value of fluid biomarkers and comment on the potential for zoonotic transmission of chronic wasting disease (CWD) from cervids (e.g., deer, elk) to humans.

This review article was conducted following a search of the published literature using several key words (Appendix A). Articles were reviewed and selected based upon clinical diagnostic relevance and the scope of this article.

## 2. Background

Human prion diseases are commonly divided into three etiologic categories: sporadic (85–90%), genetic (10–15%), and acquired (<1%). Sporadic Creutzfeldt–Jakob disease (sCJD) represents the majority of prion diseases with several distinct subtypes that are classified according to the genotype of the prion protein (*PRNP*) gene at codon 129 and the molecular properties of PrP^Sc^ on Western blot [1]. The codon 129 *PRNP* genotype may be homozygous or heterozygous for methionine (M) or valine (V). They are further classified based on the size and electrophoretic mobility of the protease-resistant core fragment into either type 1 or type 2, with some cases demonstrating both types [2]. This nomenclature has resulted in six subtypes: MM1/MV1 (considered as the same strain), VV1, VV2 (ataxic variant), MV2, MM2 (cortical variant), and MM2 (thalamic variant, also referred to as sporadic fatal insomnia (sFI)). These subtypes disproportionately affect different anatomic locations of the brain [2,3], leading to presentations with various clinical features, diagnostic testing, and imaging findings [4]. In addition to sCJD and sFI, there is a third sporadic prion disease, referred to as variably protease sensitive prionopathy (VPSPr) that demonstrates different Western blot characteristics based on partial protease K sensitivity, unique neuropathologic features, and atypical clinical characteristics [5].

Genetic prion diseases, representing 10–15% of cases, include genetic Creutzfeldt–Jakob disease (gCJD), Gerstmann–Sträussler–Scheinker disease (GSS) and fatal familial insomnia (FFI), all of which are linked to mutations in the *PRNP* gene. To date, more than 50 different mutations of *PRNP* have been linked to prion diseases with familial predisposition [6]. Different mutations in the *PRNP* gene can produce varying clinicopathological phenotypes in different individuals within or across families with the same mutation [7,8,9,10]. Other genetic factors, such as the polymorphism at codon 129 in cis with the mutation, can significantly affect clinicopathologic characteristics, as seen in gCJD (D178N-129V) and FFI (D178N-129M), which both share the same point mutation but differ in the codon 129 polymorphism on the mutated allele [11].

Acquired prion diseases, including iatrogenic CJD (iCJD), kuru, and variant CJD (vCJD), represent the most infrequent cause of prion disease but remain important for public health reasons. Kuru is a virtually extinct form of human prion disease affecting the Fore linguistic group of Papua New Guinea that was transmitted via ritualistic endocannibalism. Variant CJD is associated with ingestion of meat infected with bovine spongiform encephalopathy (BSE) and its incidence has diminished in recent years. Iatrogenic CJD is rare, though cases continue to be reported due to prior exposure and prolonged incubation periods and can occur from exposure to cadaver derived human pituitary growth hormone (HGH) and pituitary gonadotropin administration, dura mater allografts, neurosurgical instrumentation, corneal transplants, and blood transfusions (NB: three confirmed cases, only occurring from exposure to individuals infected with vCJD) [12]. Scenarios that may lead to cases of iCJD are believed to be well controlled due to current infection control practices [12].

## 3. Clinical Phenomena

Prion diseases are difficult to clinically differentiate from other neurodegenerative diseases, making it hard to determine when further investigation is warranted given their relative rarity. The incidence of CJD is approximately one to two cases per million individuals per year, worldwide [13]. The average annual incidence among those ≥65 years of age is 5.9 per million in the United States. Cases younger than 30 years of age are extremely rare and are primarily attributed to exogenous factors or genetic mutations [14]. Risk factors for sCJD include increasing age (mean age of onset at 63) and methionine homozygosity at *PRNP* codon 129. Other potential risk factors have been reported, but lack reliably replicated data.

Initial symptoms of prion diseases are usually non-specific, shared among a range of other neuropsychiatric conditions. One study looked at the initial symptoms in 114 individuals with probable or possible sCJD. The most common initial symptoms involved cognitive dysfunction (40%), with memory loss being the most prominent (45%), followed by aphasia (13%) and frontal/executive dysfunction (13%). Cerebellar (22%), constitutional (21%), and behavioral symptoms (20%) occurred with a similar frequency, while sensory (9%), motor (9%), and visual (7%) symptoms occurred less often [15]. The typical symptoms and disease course seen in sCJD includes rapidly progressive dementia, gait disturbance and limb ataxia, myoclonus, and akinetic mutism, followed by death. Prodromal constitutional symptoms, extrapyramidal signs, pyramidal signs, and visual disturbances are likely common, though it is difficult to ascertain and quantify because of recollection bias. Criteria for the clinical antemortem diagnosis of CJD are listed in Table 1. Although initial criteria that relied on clinical symptoms, cerebrospinal fluid (CSF) 14-3-3 protein results and electroencephalogram (EEG) findings lacked specificity for early stage prion disease, the currently used CSF real time quaking induced conversion (RT-QuIC) and brain magnetic resonance imaging (MRI) have significantly improved clinicians’ ability to diagnose prion disease earlier in the disease course.

## 4. Markers for Rapid Neurodegeneration

Diagnostic laboratory testing, neurodiagnostic testing, and brain imaging are useful in the diagnosis of prion disease but can be limited in specific subtypes. Additionally, many CSF biomarkers are non-specific for prion disease and are, thus, more useful as an adjunct diagnostic test, rather than a disease specific diagnostic test. The conventional diagnostic work-up for suspected prion disease includes cerebrospinal fluid testing, EEG, MRI, and potentially, a pre-mortem brain biopsy, if investigating an alternative, treatable pathology.

CSF studies have been heavily investigated regarding diagnostic utility in prion disease. The CSF profile in prion disease is typically acellular, with a normal glucose and ~40% of patients have elevated CSF protein [17]. CSF biomarkers that have been studied in prion disease primarily include 14-3-3, total tau, phosphorylated tau, neurofilament light chain, neuron-specific enolase, alpha-synuclein and to a lesser extent, S100B and thymosin B4. CSF 14-3-3 and total tau are the most commonly utilized neurodegenerative markers for prion disease in clinical practice within the United States. However, many of these biomarkers are not elevated throughout the disease course and are non-specific to prion disease.

Like other surrogate markers of neurodegeneration, CSF 14-3-3 is a marker of brain cell death and is not specific for prion disease [18]. With the advent of improved diagnostic tests for prion disease, its usefulness in countries that have access to brain MRIs and RT-QuIC is questionable and it is useful only as an adjunct biomarker rather than a disease specific diagnostic test. The sensitivity of 14-3-3 has most commonly been reported as between 85% and 95% [19,20,21,22,23,24,25], with a specificity anywhere from 40% to 100% [19,21,24,25,26,27,28,29] The sensitivity of 14-3-3 in cases of possible gCJD or the MM2 and MV2 variants of sCJD may be as low as 60% [21,23,30,31]. The moderate sensitivity, but poor specificity is likely due to its elevation in a number of different neurologic diseases, including herpes simplex encephalitis, hypoxic brain damage, atypical encephalitis, intracerebral metastases, metabolic encephalopathy, progressive dementia of unknown cause, vascular dementia, Lewy-body dementia (LBD), and Alzheimer’s disease [18,21,24,32,33]. False-positives can occur with traumatic or bloody lumbar punctures [28]. Given the low prevalence of prion disease and the low specificity of CSF 14-3-3, most positive results represent false positives. However, when other clinical features are suggestive of prion disease, a positive test does increase the probability of CJD [25].

Tau has similar diagnostic significance, though slightly improved accuracy compared to 14-3-3 with a sensitivity of 67–91% and specificity of 67–95% [20,21,22,28,29,34,35,36]. Tau can be helpful as a screening test, as almost all individuals with sCJD have total tau levels >500 pg/mL in their CSF, 90–95% have total tau >1150 pg/mL, and 80–90% have total tau levels >2500 pg/mL [28]. As such, a total tau <500 pg/mL may be helpful in ruling out sCJD [29]. Investigations have also been made into the diagnostic significance of the ratio of total tau to phosphorylated tau. An elevated ratio of total tau to phosphorylated tau levels has a specificity of 94–97% with a sensitivity ranging from 75–94% for CJD [20,37,38,39].

Recently, total tau levels have been investigated as a potential prognostic marker in prion disease in both the CSF and serum. Results are mixed as to whether plasma and CSF tau levels correlate diagnostically [40,41,42]. In one study, plasma tau and CSF t-tau were found to be significantly associated with survival in a subtype-specific manner. Plasma tau levels correlated with survival in the VV2 subtype, while CSF tau levels correlated with survival in sCJD VV2 and MM1/MV1 subtypes [40]. Another study, which reviewed 188 patients with probable or definite sCJD, found that CSF and plasma tau levels were significantly associated with survival time in patients with sCJD. Other biomarkers which were less strongly associated with survival included plasma neurofilament light (NfL) levels, CSF NfL levels, t-tau:p-tau ratio, 14-3-3, and neuron-specific enolase levels [42].

Tau may also have a role in differentiating prion disease from Alzheimer’s disease, one of the most clinically similar mimickers. CSF tau was 41 times higher in CJD than in normal controls, compared to 3.1 times higher in AD [43]. Another tau related biomarker, non-phospho-Tau (non-P-Tau), was found to be significantly elevated in CJD (3683 ± 3599 pg/mL) compared to AD (148 ± 219 pg/mL) and neurological controls (62 ± 40 pg/mL). Non-P-Tau also significantly improved differentiation of CJD from AD (99%) compared to total-Tau (90%), P-Tau (62%) and 14-3-3 (91%) [44].

Several studies have also reported elevated concentrations of serum tau in sporadic Creutzfeldt–Jakob disease [17,20,24]. When comparing sCJD to healthy controls, one study reported a sensitivity of 91% and a specificity of 83% [24], while another study reported a sensitivity of 84.6% and specificity of 96.2% [17]. However, when compared to a control group consisting of rapidly progressive dementia, diagnostic accuracy (AUC 0.722–0.837) was lower than that of CSF tau and 14-3-3 [20,23,25]. Despite this, serum tau has been reported to have increased diagnostic utility compared to serum NfL and may be useful given the relative ease in obtaining serum compared to CSF and the moderate diagnostic accuracy in differentiating clinically relevant neurological pathologies.

Neurofilament light chain (NfL) has been found to be elevated in cerebrospinal fluid (CSF) and blood in neurological diseases associated with axonal injury or degeneration [45,46,47,48]. The diagnostic potential of CSF NfL has been studied in several neurodegenerative disorders, including Alzheimer’s disease (AD) [49,50,51,52], amyotrophic lateral sclerosis (ALS) [53,54,55], multiple sclerosis (MS) [47], frontotemporal dementia (FTD) [56,57,58,59] and sporadic Creutzfeldt–Jakob disease (sCJD) [60,61]. CSF NfL has been reported to be significantly elevated in sCJD compared to other neurodegenerative disorders (AD, Lewy body dementia, FTD, vascular dementia) and MCI [62].

Several studies have reported excellent diagnostic accuracy of NfL in patients with prion disease (AUC 0.93) and sCJD (AUCs > 0.99) [41,62,63] when compared to normal controls. Additionally, CSF NfL also showed a higher diagnostic value than t-tau (AUC 0.84 vs. 0.72) in differentiating atypical prion disease from other rapid neurodegenerative dementias [64]. However, when compared to clinically similar neurological pathologies, such as Alzheimer’s disease (0.77 and 0.98) [41,64], rapidly progressive dementia (0.86–0.89) [65], neurodegenerative dementia (0.93) [64] and neurological diseases with dementia syndromes (0.45 and 0.90) [41,62], the AUCs of CSF NfL were significantly decreased (AUC 0.45–0.98) compared to CSF t-tau (AUC 0.85–0.92) and 14-3-3 (AUC 0.71–0.91) [40,41,64,65]. The superior accuracy of CSF markers in studies comparing prion diseases to clinically relevant pathologies suggest limited diagnostic utility of CSF NfL.

Given the invasive nature of acquiring CSF samples, serum NfL has been recently evaluated as a diagnostic marker in prion disease. Studies have reported sensitivity and specificity comparable to CSF markers, with a sensitivity of 100% and specificity of 85.5% when compared to healthy control groups [61,66]. However, the diagnostic accuracy decreases significantly when attempting to differentiate prion disease from rapidly progressive dementia (AUC 0.497–0.724) and was found to be less accurate than serum tau (AUC 0.722–0.837), as well as CSF tau and 14-3-3 [40,41,67]. Serum NfL values have also been investigated as a potential marker for functional impairment [42,66,67] and survival [40,42,62] in prion disease, with mixed results. However, one study did find an elevation in CSF and plasma NfL, though still within normal limits, in an asymptomatic mutation carrier of genetic prion disease, indicating potential as markers of proximity to illness onset [68].

Alpha-synuclein, commonly known for its aggregation into Lewy bodies in synucleinopathies, is another promising CSF biomarker currently being investigated. One study of 203 sCJD patients reported a sensitivity of 98% and a specificity of 97% when using a cut-off of 820 pg/mL for t-α-synuclein (total alpha-synuclein) with mean values of 324 ± 214 in non-CJD and 8906 ± 7790 in sCJD cases [69]. Another study reported 94% sensitivity and 96% specificity when diagnosing sporadic Creutzfeldt–Jakob disease using a cutoff value of 680 pg/mL [70]. Recently, p-α-synuclein (phospho-serine-129 α-synuclein) in addition to t-α-synuclein, was evaluated in a cohort of samples composed of neurological controls, sCJD, PD, and DLB. T-α-synuclein and p-α-synuclein were specifically elevated in sCJD compared to other disease groups [71]. Limitations of CSF α-synuclein are that it can be falsely positive in the presence of peripheral blood [69] and has limited use in FFI and GSS. Additionally, given that CSF α-synuclein has only been investigated by a single group, confirmatory studies are needed.

Other surrogate markers of neurodegeneration may be helpful in a diagnostic workup, but have varying sensitivities and specificities for prion disease [72], as listed in Table 2. Neuron specific enolase (NSE), a marker of brain cell death, is also elevated in acute stroke. Sensitivity ranges from 53–80% with a specificity of 83–98% [29,73,74]. S100B has a reported sensitivity ranging from 78–94%, with specificity between 81–87% in sCJD [27,75]. Sensitivity is markedly decreased in FFI (20%) and GSS (50%), though it has been reported to be as high as 92% in gCJD [76]. In one study, S100B was found in all 12 individuals with confirmed MM2 subtype of sCJD [77]. Thymosin β4 has not been well studied, but one paper did report an estimated sensitivity of 100%, with specificity of 98.5% when looking at 21 sCJD cases [78].

Investigations into the use of multiple combined biomarkers (e.g., diagnostic panel) in improving diagnostic accuracy have shown positive results. The combination of total tau and 14-3-3 have shown the most promise, with sensitivity ranging from 84–86% and specificity of 57–96%. The addition of S100B or NSE to this combination further increased the sensitivity to over 93%. However, sensitivity was low (50 to 85%) for the MV2 subtype of CJD [20,21]. There are mixed findings on the elevation of non-specific biomarkers throughout the course of the disease. Total tau and 14-3-3 have been found to peak midway in one study [87,88], while another study found progressively increasing levels in total tau, S100B, and NSE throughout the disease course [89]. Regardless, these markers do not appear to be drastically elevated early on, which could potentially obscure a prompt diagnosis. Currently, there is no generally accepted consensus on the combined use of multiple CSF biomarkers in the diagnosis of prion disease.

## 5. Non-Laboratory Based Diagnostic Tests for Prion Disease

Periodic short-wave complexes (PSWC) are the typical EEG finding commonly associated with sCJD. PSWCs are observed in 67–95% of patients with sCJD, with a sensitivity of ~65% and specificity of ~90%. PSWCs are characterized by the following features: strictly periodic cerebral potentials, the majority with a duration of 100 to 600 milliseconds and an intercomplex interval of 500 to 2000 milliseconds, generalized and/or lateralized complexes and at least five repetitive intervals with a duration difference of <500 milliseconds required to exclude semiperiodic activity. Though PSWCs have a relatively high specificity in sCJD, they can be seen in other neurologic etiologies and are not continuously present, usually occurring late in the disease course [79]. PSWCs have also been reported in patients with Alzheimer’s disease, vascular dementia, Lewy body disease, and voltage-gated potassium channel complex antibody encephalitis [90,91]. They are not found in patients with vCJD, kuru, Gerstmann–Sträussler–Scheinker syndrome (GSS), or fatal familial insomnia (FFI) and in some molecular subtypes of sCJD, but are occasionally found in patients with genetic CJD (gCJD) [79]. However, EEG remains an important neurodiagnostic test in prion disease, primarily for ruling out more common pathologies and can be an important clinical clue if PSWCs are found.

MRI has found increasing utility in diagnosing prion disease with the additional benefit of assisting in differentiating more common etiologies. Conventional MRI criteria for prion disease consists of high signal abnormalities in the caudate nucleus and putamen or at least two cortical regions (excluding frontal lobe cortex) in either DWI or FLAIR [80]. Attenuation of the signal on apparent diffusion coefficient (ADC) maps can additionally be helpful in differentiating CJD from other rapidly progressive dementias [92]. In one study, MRI was found to have a sensitivity of 98% and specificity of 93% in sCJD [29]. Additionally, MRI predicted the correct diagnosis in 14 out of 15 cases that had negative 14-3-3, NSE, and T-tau results [29]. The pulvinar sign, or “hockey-stick sign,” (isolated hyperintensity in the pulvinar nucleus of the thalamus on FLAIR, T2, or DWI) showed a sensitivity of 78% and specificity of 100% [93,94] and is specific to variant CJD. MRI findings typical of prion disease can be mimicked by pathologies such as toxic metabolic encephalopathies, progressive multifocal dementia, autoimmune encephalitis, CNS lymphoma, vasculitis, and infectious etiologies, among others [95,96].

**Figure 1:**
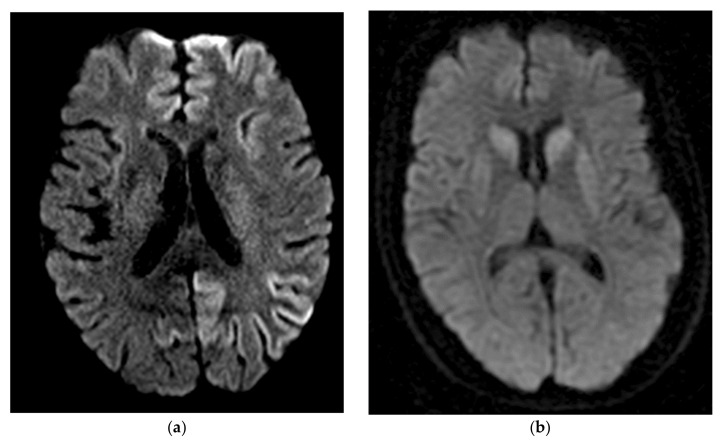
Brain MRI findings in prion disease. (**a**) Axial DWI imaging showing a widespread cortical restricted diffusion pattern typically found in sCJD MM1; (**b**) Axial DWI imaging showing restricted diffusion in the bilateral basal ganglia typically seen in sCJD VV2 (ataxic variant).

Recently, the development of new MRI criteria has exceeded the sensitivity and specificity of typical CSF biomarkers, approaching equivalence to RT-QuIC (described below). In one study, diffusion MRI was reported to have a sensitivity of 94.7% and specificity of 90%. These results utilized a newly proposed diagnostic criterion requiring a score of at least two on a four-point scale that graded DWI/ADC diffusion restriction on MRI in at least one of the following brain regions, excluding the limbic structures and the cerebellum: cortex of frontal, parietal, occipital, and temporal lobes, caudate, putamen, and thalamus. Diagnostic sensitivity reached 100% when both diffusion MRI and second-generation RT-QuIC were considered together [81]. Though from a single study that requires replication, these results support the improved clinical utility of MRI/DWI in diagnosing prion disease. As sCJD molecular subtypes have neuroanatomic localization patterns, brain MRI can be used to help differentiate sCJD molecular subtypes [97,98], as shown in Figure 1. Diagnostic accuracy of the sCJD molecular subtype can be increased if the *PRNP* codon 129 polymorphism is known.

## 6. Prion Specific Assays

**Figure 2:**
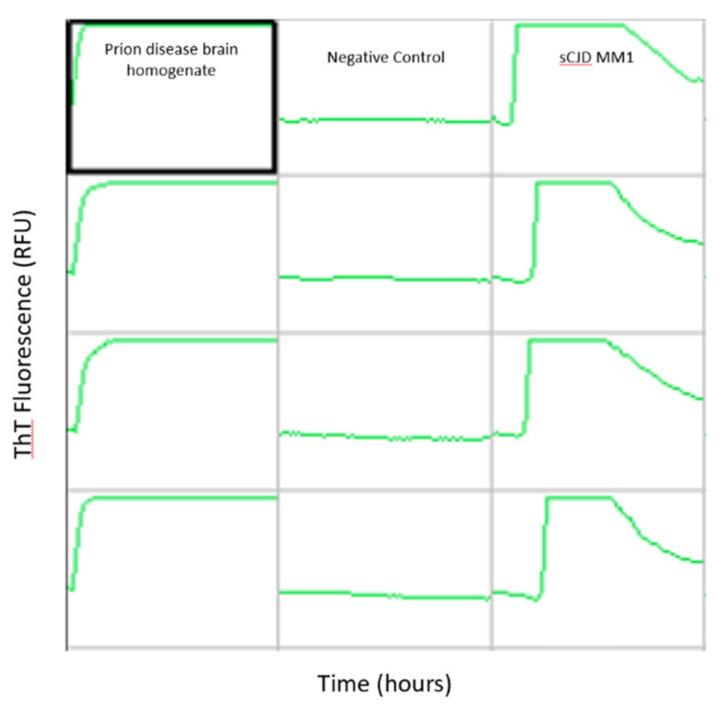
Real-time quaking-induced conversion reactions seeded with prion disease brain homogenate, and cerebrospinal fluid from a negative control and a positive result from a patient with the MM1 subtype of sporadic Creutzfeldt–Jakob disease (sCJD MM1). Each sample is run in quadruplicate, organized vertically. Thioflavin T (ThT) fluorescence, indicative of amyloid formation, is measured over time. In the positive sCJD MM1 CSF sample, ThT fluorescence initially increases due to amyloid formation, followed by a characteristic decline thought to be due to self-quenching.

CSF real-time quaking-induced conversion (second generation RT-QuIC) is the first laboratory based, prion-specific, clinically available test that does not require brain tissue. It was first made available routinely in the United States in 2015 and was added to the CDC’s diagnostic criteria for prion disease in 2018. As shown in Figure 2, RT-QuIC exploits the autocatalytic template directed protein misfolding nature of prions to propagate and aggregate when exposed to recombinant prion protein, resulting in amyloid formation. The sensitivity of RT-QuIC ranges from 90.3–97.2%, with a specificity of 98.5–100% across all prion diseases [82,83,84]. In sCJD, sensitivity and specificity have been reported as high as 97 and 100%, respectively [85]. In addition to improved diagnostic accuracy, the sensitivity of RT-QuIC remains high throughout the disease course as opposed to some of the non-specific CSF biomarkers. RT-QuIC does have limitations in that elevated white cell count, erythrocytes and protein can interfere with the results [82,99]. Very low levels of prions may go undetected and sensitivity is decreased for FFI, sFI, VPSPr, GSS, and the VV1 and MM2 subtypes of sCJD likely due to strain (conformer) differences [84]. Additionally, RT-QuIC takes days to complete, though investigations into the optimization of RT-QuIC have shown promise in reducing time constraints [100]. Current diagnostic criteria do not make a distinction between second generation RT-QuIC (IQ-CSF) and first generation RT-QuIC, which has reduced diagnostic accuracy. Unfortunately, RT-QuIC is largely unavailable in countries without prion disease surveillance programs [101].

Western blotting for PrP^Sc^ and immunohistochemistry were previously the only prion-specific assay prior to the introduction of RT-QuIC and required a sample of brain tissue for analysis (i.e., pre-mortem brain biopsy). While technically required for definitive diagnosis, this is largely unnecessary in the era of RT-QuIC unless attempting to ascertain an alternative, treatable diagnosis. Otherwise, definitive diagnosis should be deferred until autopsy with focus on supportive and symptomatic treatment. There are no effective disease-modifying treatments that have been identified for prion diseases and neurosurgery carries exposure risks. Additionally, Western blot analysis of brain biopsies has a poor sensitivity of 20–60% as prion deposition in the brain can occur at various sites and is dependent on the type of tissue collected during the biopsy [86]. For example, tissue from a typical biopsy site (e.g., frontal cortex), obtained in sporadic fatal insomnia, would be expected to have negative results as prion deposition is scant and the thalamus is primarily affected in this prion disease.

Diagnosis using tissue samples outside of the CNS are being investigated, but currently lack sufficient evidence to be of clinical diagnostic utility, with the exception of tonsil biopsy in vCJD [102,103,104,105,106,107,108,109]. There is also ongoing research into the use of RT-QuIC on other tissues, such as the skin [102], eyes [103], and olfactory epithelium [110]. Notably, one study did report that the combination of CSF and olfactory mucosa RT-QuIC showed 100% diagnostic sensitivity and specificity in sCJD [111]. Another study reported 89% sensitivity and 100% specificity of RT-QuIC in skin punch biopsies of 35 patients with Creutzfeldt–Jakob disease (CJD), including five assessed ante-mortem [112].

## 7. Genetics

*PRNP* gene sequencing is the primary diagnostic technique in genetic prion disease. Detection of *PRNP* gene mutations can be performed by sequencing DNA from patient’s blood specimens or decedent’s unfixed autopsy tissue. All three genetic forms of prion disease are linked to *PRNP* mutations and include point mutations, octapeptide repeat insertions and deletions. Many different mutations have been linked to gCJD, though the most common worldwide is E200K. All patients with definite GSS have been found to have several different *PRNP* mutations and the D178N-129M mutation is found in all families with FFI [113]. It is important to realize that disease penetrance varies widely depending on the *PRNP* mutation [114]. In certain cases, *PRNP* gene sequencing can also be useful in differentiating gCJD from sCJD due to clinical similarities between the two etiologies. Although sCJD and iCJD are not associated with *PRNP* gene mutations, phenotyping at codon 129 may affect susceptibility as 85–95% of sCJD cases and all but one case of vCJD are homozygous at codon 129 compared to 49% of the normal population [115]. However, the codon 129 polymorphism is primarily investigated in research studies and is not currently used in the diagnostic work-up of prion disease. However, there may be a role in stratifying patients by codon 129 polymorphism in clinical treatment trials [116].

## 8. Atypical Prion Disease

In the era of RT-QuIC, many of the challenges in the diagnosis of prion disease have been simplified. RT-QuIC has extraordinary sensitivity and specificity in prion disease, especially when combined with a typical presentation and positive MRI findings. Though RT-QuIC and newer MRI criteria have substantially improved diagnostic accuracy, limitations still exist. MRI findings are clinician-dependent and may be missed [117], while RT-QuIC has decreased sensitivity in certain genetic and atypical sporadic prion disease. The prion disease subtypes that have decreased sensitivity with RT-QuIC include FFI, GSS, sFI, VPSPr and the VV1 and MM2 subtypes of sCJD. These prion diseases are additionally challenging to diagnose due to atypical presentations that may not overtly raise the suspicion of prion disease or match the classic CJD phenotype that is usually associated with MM(MV)1 sCJD. The utility of clinically relevent diagnostic tests are listed in Table 3.

First reported in 2008, variably protease-sensitive prionopathy (VPSPr) illustrates an ongoing challenge in the recognition and diagnosis of prion diseases. To date, more than 40 cases of VPSPr have been identified [118,119,120,121,122,123]. The presentation of VPSPr more closely resembles atypical dementia, such as normal pressure hydrocephalus, dementia with Lewy bodies, or frontotemporal dementia than sCJD [124]. Several commonly used diagnostic tests are typically unremarkable in VPSPr, including normal findings or diffuse slowing on EEG, lack of diffusion restriction on MRI and normal CSF 14-3-3 [122]. RT-QuIC has a sensitivity of approximately 66% in VPSPr [84]. Interestingly, VPSPr is observed in all three genotypes at residue 129 of *PRNP*. However, this is not currently included in the diagnostic work-up.

Fatal familial insomnia (FFI) is a genetic prion disease with a distinct, but a difficult to recognize clinical presentation and largely unremarkable diagnostic testing. The median age of onset in FFI is 56 with a mean disease duration of 13 months. Symptoms include a characteristic progressive insomnia with loss of normal circadian sleep-activity pattern resulting in a dream-like state while awake [125], mental status and behavioral changes, but rarely presents with features typical of dementia [113,126,127,128]. FFI also has some unique symptoms including dysautonomia (hyperhidrosis, hyperthermia, tachycardia, hypertension) and endocrine disturbances (increased cortisol, decreased ACTH, loss of diurnal variations in growth hormone, melatonin and prolactin) [113,129,130]. In addition to the atypical presentation of FFI, many commonly used biomarkers and neurodiagnostic tests are unhelpful. CSF 14-3-3 is not detectable [131], EEG does not show PSWCs and brain MRI is typically normal. FDG-PET and sleep studies may have some clinical relevance. FDG-PET has been reported to show decreased glucose uptake in the thalamus [132,133,134] and sleep studies show abnormal disruption in sleep architecture with decreased total sleep time [135]. However, the primary diagnostic technique in FFI is genetic testing given that all cases are associated with the D178N-129M *PRNP* gene mutation. The sporadic version of fatal insomnia, sFI, can similarly be difficult to diagnose, especially with the absence of a genetic mutation. Recognition of the clinical syndrome with abnormal polysomnography and/or thalamic hypometabolism on brain FDG-PET with methionine homozygosity at codon 129 of *PRNP* can be helpful in making the antemortem diagnosis of this rare prion disease. In one study, only two of thirteen cases of sFI were reported to be RT-QuIC positive [136].

GSS is another genetic prion disease with atypical features. The age of onset occurs in the mid-40s, significantly younger than in sCJD, and primarily presents with cerebellar symptoms rather than subacute, progressive dementia, typically spread out over several years duration. As in FFI, the CSF is bland, EEG does not show PSWCs and brain MRI is not sensitive or specific. CSF RT-QuIC is positive in about one third of GSS cases [84]. However, since GSS cases are familial and all patients with definite GSS have been found to have *PRNP* mutations, demonstration of *PRNP* gene mutations remains the primary diagnostic test. The most common mutation in GSS, P102L, was found in the descendants of the original Italian family described by Gerstmann, Sträussler, and Scheinker [2,3,4].

The MV2 molecular subtype of sCJD accounts for ~10% of sCJD cases. The typical disease course occurs over ~17 months, presenting with ataxia, progressive dementia, and prominent psychiatric features [31]. PSWCs are rare [21,23,30,31] and 14-3-3 has a reported sensitivity ranging from 30–76%. In one study looking specifically at the MV2 subtype, the combination of total tau and 14-3-3 resulted in a sensitivity of 89%, while MRI had a sensitivity of 90%, showing a high frequency of thalamic hyperintensity. PSWCs had a sensitivity of 8% and were found in only two of the twenty-six patients. The same study reported that sporadic CJD was not initially proposed in any of the cases and was not suspected until 8 months after disease onset, highlighting the challenge of diagnosing atypical prion disease [137]. Fortunately, CSF RT-QuIC has a sensitivity of 92.5% for sCJD MV2 [84].

The VV1 variant of sCJD is the rarest sCJD molecular subtype, accounting for ~1% of sCJD cases. Based on a study of nine patients with confirmed pathology, unique features of VV1 include a younger age of onset (~44 years-of-age) with a more prolonged disease course (median of 21 months). None of the individuals showed PSWCs on EEG, though all had elevated CSF 14-3-3. CSF RT-QuIC is typically negative or indeterminate in sCJD VV1 [84]. MRI findings were notable for increased cortical signal, while only two had MRI findings of increased signal in the basal ganglia [138].

Variant CJD has diminished in recent years with only 232 total cases reported worldwide beginning in 1996. Cases have steadily declined over the last decade and there have not been any reported cases worldwide in the past year. Both CSF tau and 14-3-3 had mixed clinical relevance [139,140] and RT-QuIC has markedly decreased sensitivity for vCJD, with one study reported a sensitivity of 25% [83]. EEG was found to be abnormal in ~70% patients, but PSWCs are generally absent [141,142]. MRI typically shows signal hyperintensity in the pulvinar nucleus of the thalamus (pulvinar sign) or in both the pulvinar and dorsomedial thalamus (hockey stick sign) [93,94]. Unlike other prion diseases, vCJD can be reliably diagnosed by examination of tonsillar tissue via Western blot analysis, which has high sensitivity and specificity [104,143,144].

## 9. The Potential for Emerging Zoonotic Prion Disease

Chronic wasting disease (CWD) is a prion disease that affects white-tailed and mule deer, elk, and moose, which may have the potential for zoonotic transmission to humans. In cervids, symptoms can include significant weight loss (wasting), stumbling, exhaustion, behavioral changes, excessive salivation, difficulty swallowing, polydipsia, and polyuria [145,146,147,148]. CWD has been confirmed in at least 26 U.S. states, three Canadian provinces, South Korea, Finland, Norway, and Sweden. Transmission most likely occurs via infectious bodily fluids such as saliva, urine, and feces [149] and potentially remain infectious when transported [150].

The CWD prion has several properties that have led to new strains with variable symptoms [151,152,153], a broader range of hosts and increased zoonotic transmission potential that make it of concern for human public health [154,155]. In vitro, the CWD prion has been shown to be capable of converting the wild-type human PrP^c^, though with low efficiency or requiring protein modifications [156]. Animal studies have reported the ability of the CWD prion to infect hamsters, transgenic mice expressing hamster PrP or overexpressing mouse PrP, sheep, cattle and squirrel monkeys [157,158,159,160,161]. However, there is evidence that a human species barrier exists, as transgenic mice expressing human PrP were not susceptible to CWD by intracerebral inoculation [162,163]. Transmission of CWD has also been evaluated in macaque monkeys, animals more genetically similar to humans, with conflicting results. A 2018 study found no evidence of CWD transmission in macaque monkeys inoculated with CWD prions [164]. However, a yet unpublished study with oral or intracranial inoculation of CWD into 18 macaque monkeys presented evidence supporting the potential for zoonotic infection [165]. As of 2017, 11 macaques were available for assessment. Three displayed neurological signs, six had wasting (pre-clinical or clinical diabetes) and five had prion specific histopathologic lesions displaying PrP^Sc^ deposits or amyloid seeding. Of these five animals, two were seeded intracranially, while three were fed muscle tissue from white-tailed deer with pre-clinical or clinical CWD [165]. Given that the macaques that were inoculated via consumption of CWD infected muscle tissue developed histopathologically proven lesions, human consumption of meat from CWD infected cervids may pose a risk of zoonotic transmission. Currently in cervids, CWD is diagnosed post-mortem by ELISA and immunohistochemical analysis of brain stem or retropharyngeal lymphoid tissue. ELISA is not validated for use in elk and moose and both ELISA and IHC lack sensitivity in cases with subclinical disease [166]. Recent investigations into ante-mortem surveillance of CWD in cervids have shown promise utilizing RT-QuIC in lymphoid tissue of rectal biopsies (sensitivity 69.8%, specificity > 93.9%) [167] and ear pinna punch biopsies (sensitivity 81%, specificity 91%) [168]. If CWD were to transmit to humans, the clinical phenotype and the sensitivities of conventional diagnostic tests used to diagnose human prion disease are unknown. There are several long-term surveillance programs in individuals who have consumed confirmed CWD positive meat despite current recommendations and concern for possible transmissibility. There are almost 1000 participants in one such program by the Wisconsin Department of Health Services [169]. While there have not been any confirmed cases of human CWD, studies are concerning for the potential of zoonotic transmission to humans [170].

## 10. Conclusions

Advances in diagnostic testing have led to increasingly specific detection of prion disease. RT-QuIC, especially when combined with newly developed MRI criteria, has greatly improved diagnostic accuracy. Limitations exist, primarily in regard to the rarer and atypical prion diseases. Generally, except for RT-QuIC, CSF biomarkers are non-specific to prion disease and typically are not positive at disease onset. However, tau and alpha-synuclein are showing increasing diagnostic relevance. Additionally, tau shows promise both as a prognostic marker and in differentiating prion disease from Alzheimer’s disease. EEG remains an important neurodiagnostic test for discerning prion disease from clinically similar pathologies and the presence of PSWCs is relatively specific for the most common subtypes of sCJD. RT-QuIC should be obtained as soon as prion disease is considered. If negative, atypical prion diseases can be investigated in the right clinical context due to the lower sensitivity of RT-QuIC in these pathologies. Genetic testing can be diagnostic in many of these situations as genetic prion diseases are more prevalent than the rare, atypical sCJD subtypes. In these cases, MRI findings can be helpful in all but VPSPr and sFI. Given the clinical and anatomic similarities to FFI, polysomnography and brain FDG-PET can also be helpful in diagnosing sFI. The combination of MRI, RT-QuIC, and genetic testing effectively eliminates any diagnostic utility in obtaining pre-mortem brain biopsy, unless trying to ascertain an alternative, treatable pathology. Increasing concern for the zoonotic potential of CWD reinforces the importance of post-mortem autopsy in prion disease surveillance and continued investigation into novel ante-mortem testing modalities.

## Figures and Tables

**Table 1:** Diagnostic criteria for Creutzfeldt–Jakob disease [16].

Definite CJD Requires Neuropathological Diagnosis, Immunohistochemical Confirmation, Tissue Western Blotting for Proteinase-Resistant PrP, or the Presence of Scrapie-Associated Fibrils.
**Probable CJD** criteria are fulfilled by a neuropsychiatric disorder plus a positive real-time quaking-induced conversion (RT-QuIC) test **OR** a rapidly progressive dementia with at least 2 of the 4 following clinical symptom criteria: Myoclonus Visual or cerebellar signs Pyramidal or extrapyramidal signs Akinetic mutism **PLUS** at least one of the following diagnostic test findings: Periodic sharp-wave complexes (PSWCs) on EEG during an illness of any duration A positive 14-3-3 CSF test in a patient with disease for less than 2 years High signal in caudate/putamen on magnetic resonance imaging (MRI) brain scan or at least two cortical regions (temporal, parietal, occipital) either on diffusion-weighted imaging (DWI) or fluid attenuated inversion recovery (FLAIR) **AND** without routine investigations indicating an alternative diagnosis
**Possible CJD** is defined as a progressive dementia with at least 2 of 4 of the following criteria: Myoclonus Visual or cerebellar signs Pyramidal/extrapyramidal signs Akinetic mutism plus a duration of less than 2 years. **AND** the absence of a positive result for any of the four tests above that would classify a case as “probable” **AND** duration of illness less than two years **AND** without routine investigations indicating an alternative diagnosis
**Iatrogenic CJD** is defined as a progressive cerebellar syndrome in a patient who received human cadaver-derived pituitary hormone or sCJD with a known high-risk exposure
**Genetic CJD** is classified as definite/probable CJD plus a first-degree relative with definite/probable CJD or a neuropsychiatric disorder with a disease-specific *PRNP* mutation.

**Table 2:** Diagnostic testing in prion disease.

Test	Sensitivity	Specificity	References
Non-specific biomarkers			
CSF 14-3-3	85–95%	40–100%	[19,20,21,22,23,24,25,26,27,28,29]
CSF Tau	67–91%	87–95%	[20,21,22,28,29,34,35,36]
CSF T-tau/P-tau ratio	75–94%	94–97%	[20,37,38,39]
Serum/plasma Tau	57–91%	83–97%	[61,66,67]
CSF NfL	86–97%	43–95%	[34,61,64]
Serum/plasma NfL	93–100%	57–100%	[61,66,67]
Alpha-synuclein	94–98%	96–97%	[69,70]
S100B	78–94%	81–87%	[27,75]
NSE	53–80%	83–98%	[29,73,74]
Thymosin β4	100%	98.5%	[78]
14-3-3 + T-tau	84–86%	57–96%	[20,21]
+NSE or S100B	93%		
Neurodiagnostic Tests			
EEG	~65%	~90%	[79]
MRI	94.7–98%	90–100%	[80,81]
Prion-specific Tests			
RT-QuIC (2nd generation)	90.3–97.2%	98.5–100%	[82,83,84,85]
Brain tissue PrP^Sc^ Western blotting	20–60%		[86]

**Table 3:** Clinical Utility of Pre-mortem Diagnostic Testing in Atypical Prion Disease.

Disease	Tests with High Disease-Specific Diagnostic Utility	Tests with Lower Disease-Specific Diagnostic Utility
VPSPr	None	14-3-3, RT-QuIC, EEG, MRI
sCJD MV2	RT-QuIC, MRI	14-3-3, tau, EEG
sCJD VV1	MRI, 14-3-3	RT-QuIC, EEG
sFI	Brain FDG-PET, polysomnography, *PRNP* gene sequencing	14-3-3, tau, RT-QuIC, EEG, MRI
FFI	Brain FDG-PET, polysomnography, *PRNP* gene sequencing	14-3-3, tau, RT-QuIC, EEG, MRI
GSS	*PRNP* gene sequencing	14-3-3, tau, RT-QuIC, EEG, MRI
vCJD	Tonsil biopsy, MRI (pulvinar or hockey-stick sign)	14-3-3, RT-QuIC, EEG

## Appendix A

This review was conducted via a search of the literature based on certain key words and phrases. Articles were reviewed and selected based upon clinical diagnostic relevance and the scope of this article.
